# Molecular Composition of Staufen2-Containing Ribonucleoproteins in Embryonic Rat Brain

**DOI:** 10.1371/journal.pone.0011350

**Published:** 2010-06-28

**Authors:** Marjolaine Maher-Laporte, Frédéric Berthiaume, Mireille Moreau, Louis-André Julien, Gabriel Lapointe, Michael Mourez, Luc DesGroseillers

**Affiliations:** 1 Département de Biochimie, Université de Montréal, Montréal, Québec, Canada; 2 Pathologie et Microbiologie, Université de Montréal, Montréal, Québec, Canada; Medical College of Georgia, United States of America

## Abstract

Messenger ribonucleoprotein particles (mRNPs) are used to transport mRNAs along neuronal dendrites to their site of translation. Numerous mRNA-binding and regulatory proteins within mRNPs finely regulate the fate of bound-mRNAs. Their specific combination defines different types of mRNPs that in turn are related to specific synaptic functions. One of these mRNA-binding proteins, Staufen2 (Stau2), was shown to transport dendritic mRNAs along microtubules. Its knockdown expression in neurons was shown to change spine morphology and synaptic functions. To further understand the molecular mechanisms by which Stau2 modulates synaptic function in neurons, it is important to identify and characterize protein co-factors that regulate the fate of Stau2-containing mRNPs. To this end, a proteomic approach was used to identify co-immunoprecipitated proteins in Staufen2-containing mRNPs isolated from embryonic rat brains. The proteomic approach identified mRNA-binding proteins (PABPC1, hnRNP H1, YB1 and hsc70), proteins of the cytoskeleton (α- and β-tubulin) and RUFY3 a poorly characterized protein. While PABPC1 and YB1 associate with Stau2-containing mRNPs through RNAs, hsc70 is directly bound to Stau2 and this interaction is regulated by ATP. PABPC1 and YB1 proteins formed puncta in dendrites of embryonic rat hippocampal neurons. However, they poorly co-localized with Stau2 in the large dendritic complexes suggesting that they are rather components of Stau2-containing mRNA particles. All together, these results represent a further step in the characterization of Stau2-containing mRNPs in neurons and provide new tools to study and understand how Stau2-containing mRNPs are transported, translationally silenced during transport and/or locally expressed according to cell needs.

## Introduction

In neurons, mRNA transport is widely used to differentially regulate protein content in domains distant from the cell body [1]. Especially, mRNA transport and local translation are known to be involved in neuron development, synaptic functions and plasticity [2], [3], [4]. A current model stipulates that, along the way from nuclear export to dendritic anchoring, proteins are added or removed from the mRNP complexes in a dynamic way. It was proposed that these proteins finely control the successive steps that ensure proper expression of mRNA at specific times and space. Several combinations of mRNAs and proteins form a highly heterogeneous population of ribonucleoprotein (RNP) complexes that are linked to different forms of synaptic activity and/or plasticity [5], [6], [7]. In particular, two large families of RNPs have been suggested: mRNA particles and mRNA granules [3], [6]. mRNA particles are distinguished from mRNA granules by the absence of ribosomes. It was suggested that RNA particles might represent the observed transport mRNPs [2].

**Figure 1:**
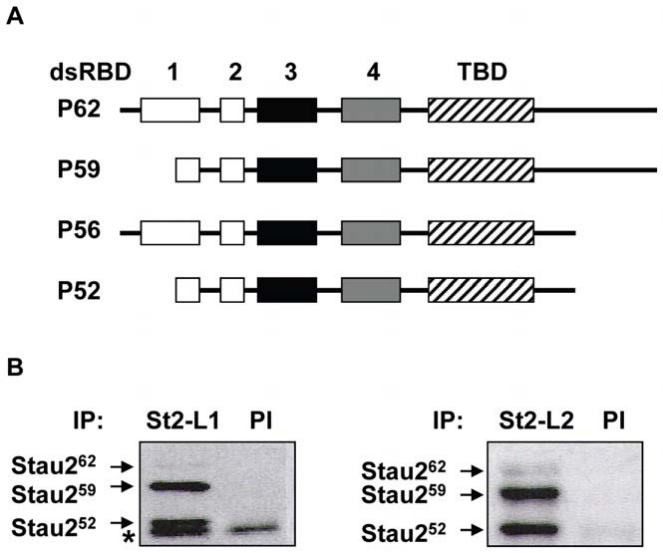
Immunoprecipitation of Stau2 isoforms. (**A**) Schematic representation of Stau2 isoforms. The *Stau2* gene generates four different isoforms of 62, 59, 56 and 52 kDa through differential splicing. Black, grey and white boxes represent double-stranded RNA-binding (dsRBD) consensus sequence having full, partial or no RNA-binding activity, respectively. Hatched boxes represent the tubulin-binding domain (TBD). (**B**) Immunoprecipitation of Stau2 isoforms from embryonic E17-18 rat brain extracts using two different polyclonal anti-Stau2 antibodies, L1 (St2-L1) and L2 (St2-L2). The specificity of these antibodies was previously reported [7]. Pre-immune (PI) sera were used as controls. The Stau2^56^ isoform is not visible in these cell extracts. * represents a non-specific IgG band.

Staufen2 (Stau2), a protein mainly expressed in brain is a well accepted player for mRNA localization [7], [8]. The *Stau2* gene expresses four protein isoforms of 62, 59, 56 and 52 kDa that are generated by differential splicing (Fig. 1) [7]. Stau2 binds double-stranded RNAs and is incorporated into mRNPs that move along microtubules in neuronal dendrites [6], [7], [8]. Interestingly, its level of expression in dendrites regulates the level of transported mRNAs showing the importance of Stau2 for mRNA transport. Likely as a consequence, neurons in which Stau2 has been down-regulated by RNAi show a reduced density of dendritic spines, associated with a change in their morphology. These phenotypes result in reduced amplitude of the miniature excitatory postsynaptic currents, a measure of synaptic transmission [9].

Stau2 was described in both the nucleus and somatodendritic compartment of the cells [7], [10]. Accordingly, Stau2 was shown to associate with nuclear factors suggesting an early role for Stau2 in mRNP assembly [11], [12]. In the somatodendritic compartment, Stau2 associates with both mRNA granules and mRNA particles. On a sucrose gradient, Stau2^62^ co-fractionates with ribosome-free particles whereas Stau2^59^ and Stau2^52^ were found in fractions that contained ribosomes [7]. However, the composition of these complexes is still largely unknown. In the work described in this paper, we immunoprecipitated Stau2-containing mRNPs and used a proteomic approach to identify Stau2-associated proteins. Several RNA-binding proteins and proteins of the cytoskeleton have been identified.

## Results

### Isolation and characterization of Stau2-containing mRNPs

In order to identify the protein content of Stau2-containing RNPs, extracts of embryonic rat brains were prepared and endogenous Stau2 was immunoprecipitated using the polyclonal anti-Stau2 antibody L1 and its pre-immune serum as control (Fig. 1). Following separation of co-immunoprecipitated proteins by SDS-PAGE, the gel was cut into bands of 3 mm and the proteins digested in-gel with trypsin. Resulting peptides were identified by mass spectrometry. In addition to Stau2, seven proteins were present in the Stau2 immunoprecipitate (Table 1 and Supplemental Table S1). Y box-binding protein 1 (YB1), polyadenylate-binding protein cytoplasmic 1 (PABPC1), heat-shock cognate protein 70 (hsc70) and heterogeneous nuclear ribonucleoprotein H1 (hnRNP H1) are RNA-binding proteins previously associated with other mRNPs, α- and β-tubulin are protein components of the cytoskeleton and RUFY3 (rap2-interacting protein X) is still poorly characterized.

**Table 1:** Proteomically identified proteins in Stau2-containing mRNPs.

Name	Peptides	Description/function	References
Staufen 2	12	Double-stranded RNA-binding protein. Mainly involved in mRNA transport	[7]
PABPC1	7	Polyadenylate-binding protein cytoplasmic 1. Mainly involved in the regulation of translation	[44]
YB1	3	Y-box 1 RNA-binding protein. Mainly involved in the regulation of translation	[36]; [55]
Hsc70	3	heat-shock cognate RNA-binding protein 70. Involved in nuclear trafficking, RNA chaperone, kinesin-mediated transport	[49]; [48]; [53]
hnRNP H1	1	heterogeneous nuclear ribonucleoprotein H1. Poly(rG)-RNA-binding protein	[56]
α-tubulin (1A, 1B)	5	Component of microtubule	[57]
β-tubulin (2b)	12	Component of microtubule	[57]
RUFY3	10	Protein interacting with rap2. Role in axonogenesis	[35]

### PABPC1, YB1, and hsc70 co-immunoprecipitate with Stau2^62^ and Stau2^59^

**Figure 2:**
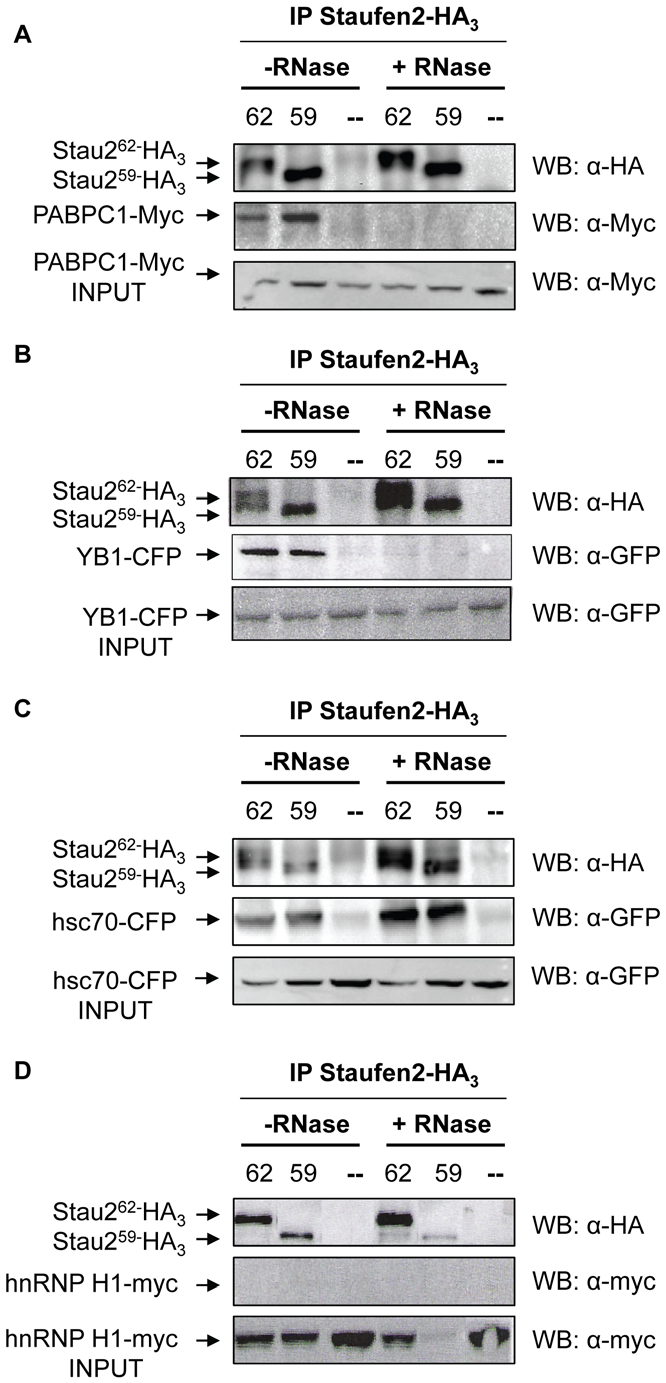
RNA-binding proteins are associated with Stau2 isoforms in mRNPs. N2A cells were mock transfected (−) or co-transfected with plasmids coding for either Stau2^59^-HA_3_ (59) or Stau2^62-^ HA_3_ (62) and plasmids coding for PABPC1-myc (**A**), YB1-CFP (**B**), hsc70-CFP (**C**) or hnRNP H1-myc (**D**) as indicated. Immunoprecipitation of Stau2-containing RNPs was performed with anti-HA antibody and the proteins detected on western blots using anti-HA, anti-myc or anti-GFP antibodies as needed. The experiments were done in the absence (-RNase) or presence (+RNase) of Microccocal nuclease to determine if the Stau2-protein association requires an RNA bridge. These results were representative of at least three experiments. Input (INPUT) of transfected proteins before immunoprecipitation is also shown to indicate that the tagged-proteins were well expressed in these cells.

It was previously shown that Stau2^62^ co-fractionated with ribosome-free mRNPs whereas Stau2^59^ and Stau2^52^ isoforms were found in heavy fractions that also contained ribosomes [7]. Therefore, we first tested whether the association between Stau2 and the identified RNA-binding proteins is specific for one Stau2 isoform or present in different complexes. In addition, we determined whether the association is direct or whether it involves an RNA intermediate. To this end, N2A cells were transfected with plasmids coding for Stau2^62^-HA_3_ or Stau2^59^-HA_3_ and tagged proteins as indicated (Fig. 2). Cell extracts were prepared and Stau2 was immunoprecipitated using anti-HA antibody, in the presence or absence of the micrococcal nuclease. Co-immunoprecipitated proteins were analyzed by western blotting using anti-myc or anti-GFP antibody as indicated. In the absence of micrococcal nuclease, PABPC1-myc, YB1-CFP and hsc70-CFP were found in Stau2^62^-HA_3_ and Stau2^59^-HA_3_ immunoprecipitates indicating that they are present in the same complexes as Stau2-HA_3_ isoforms (Fig. 2 A–C). In contrast, hnRNP H1-myc was not detected (Fig. 2D). When micrococcal nuclease was added to the cell extracts before immunoprecipitation, Stau2^62^-HA_3_ and Stau2^59^-HA_3_ interaction with hsc70-CFP was still observed (Fig. 2C) whereas interactions with PABPC1-myc and YB1-CFP were lost (Fig. 2 A,B). These results show that several RNA-binding proteins are present in the same complexes as Stau2 isoforms and suggest that only the Stau2/hsc70 interaction involved direct protein-protein interaction.

### Direct interaction between hsc70 and Stau2^62^

**Figure 3:**
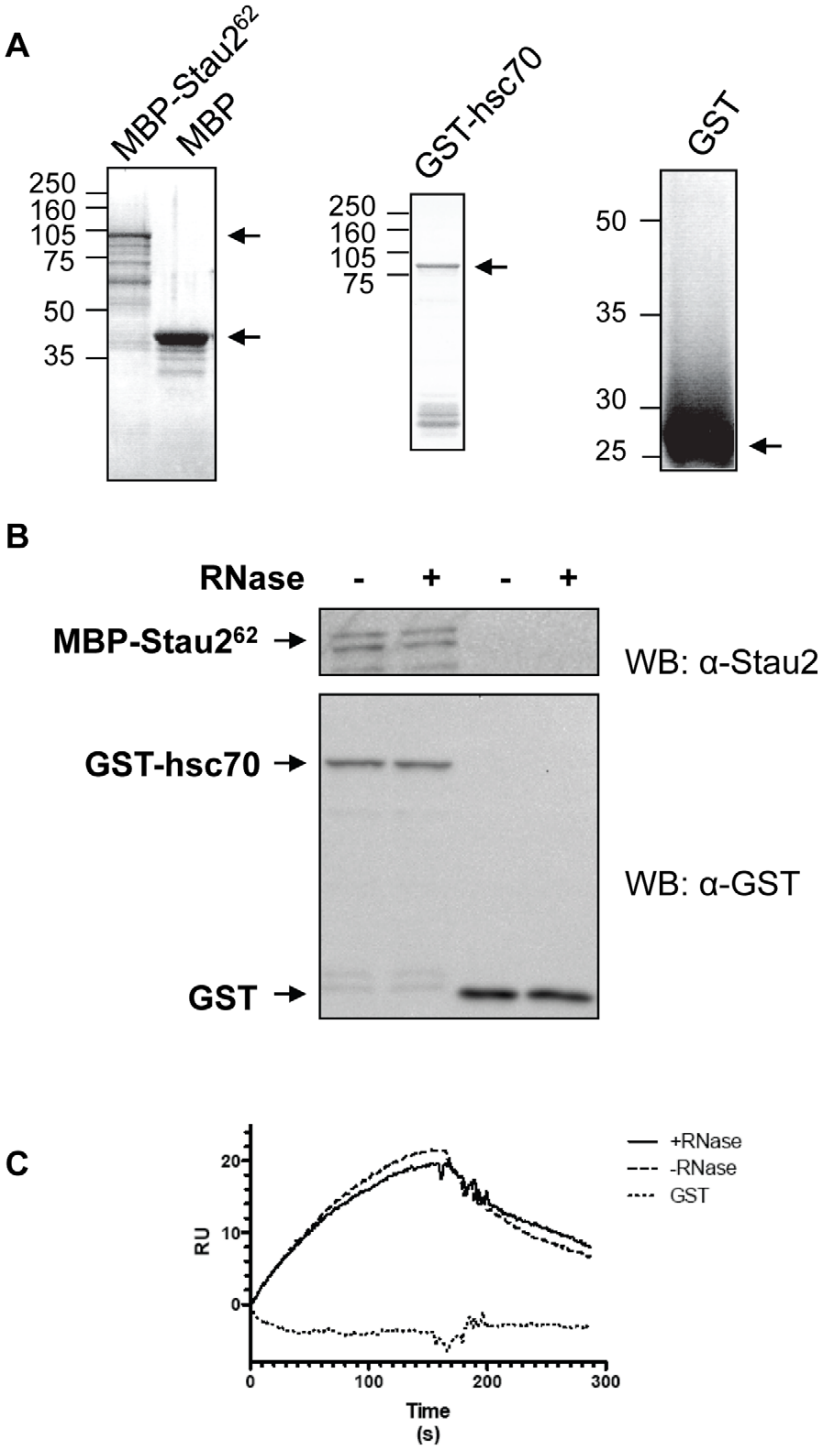
MBP-Stau2^62^ binds GST-hsc70 through protein-protein interaction. To confirm the RNA-resistant interaction between Stau2 and hsc70, bacterially expressed proteins were purified (**A**) and GST-pull down (**B**) and surface plasmon resonance SPR (**C**) assays were performed in the presence or absence of RNase A. (**A**) MBP-Stau2^62^, MBP, GST-hsc70 and GST were purified on amylose and glutathione-Sepharose-4B affinity columns, respectively, and eluted proteins were analyzed by SDS-PAGE and Coomassie brilliant blue staining. (**B**) GST and GST-hsc70 were fixed on a glutathione-Sepharose-4B affinity column and MBP-Stau2^62^ was loaded in the presence (+) or absence (−) of RNase A. After several washing, proteins were eluted from the columns and detected by western blotting using anti-Stau2 and anti-GST antibodies, respectively. (**C**) MBP and MBP-Stau2^62^ were immobilized on different lanes of a SPR sensor chip. GST-hsc70 or GST were injected for 3 minutes over the surfaces in the presence or absence of RNase and then buffer alone was injected for 2.5 min to monitor protein dissociation rate. The resulting resonance units (RU) were measured during the association and dissociation phases. The baseline obtained with the MBP-coupled reference surface was subtracted from the sensorgram obtained from the MBP-Stau2^62^-coupled surface and a typical result is shown.

To confirm the interaction between hsc70 and Stau2 at the protein level, we performed two in vitro binding assays, GST-pull down and surface plasmon resonance (SPR). To this end, bacterially expressed GST, maltose-binding protein (MBP), GST-hsc70 and MBP-Stau2^62^ fusion proteins were affinity purified (Fig. 3A). For the pull down assay, GST as a negative control and GST-hsc70 were attached to glutathione columns. Their ability to bind MBP-Stau2^62^ in the presence or absence of RNase A was tested by western blotting. In contrast to GST that failed to pull-down MBP-Stau2^62^, the GST-hsc70 fusion protein was able to bring down MBP-Stau2^62^ even in the presence of RNase A (Fig. 3B). Similarly, MBP and MBP-Stau2^62^ were fixed to the SPR sensor chip surface. Then GST and GTS-hsc70 were flowed over the SPR chip surface and their interaction with the immobilized proteins was monitored in real time. The resulting sensorgrams indicate that a direct interaction occurred between GST-hsc70 and MBP-Stau2^62^ even in the presence of RNase A (Fig. 3C). These *in vitro* binding studies confirm the ability of hsc70 to interact with Stau2, independent of RNA.

### ATP modulates the interaction between hsc70 and Stau2^62^

**Figure 4:**
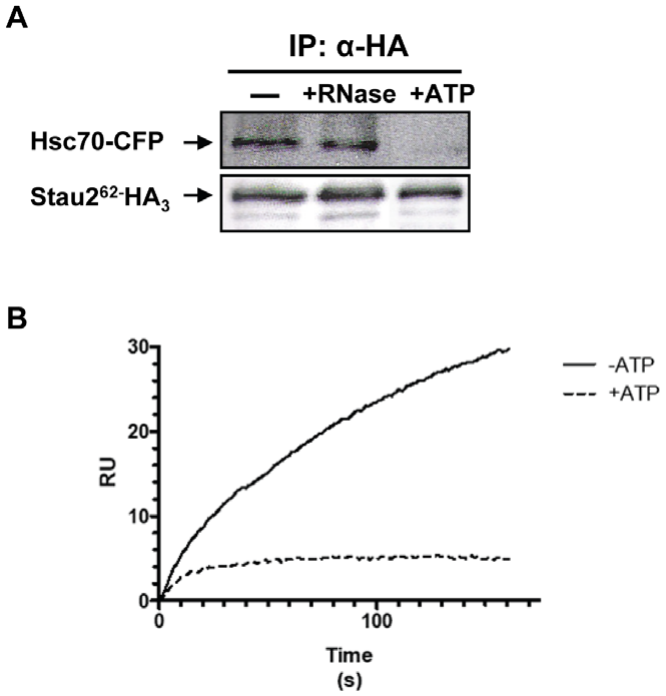
The interaction between Stau2^62^ and hsc70 is abolished in the presence of ATP. (**A**) N2A cells were co-transfected with plasmids coding for Stau2^62-^ HA_3_ and hsc70-CFP as done for figure 2. Immunoprecipitation of Stau2-containing RNPs was performed with anti-HA antibody and the proteins detected on western blots using anti-HA or anti-GFP antibodies as needed. The experiment was done in the absence (−) or presence of either Microccocal nuclease (+RNase) or ATP (+ATP). These results are representative of at least three experiments. (**B**) As done for figure 3C, MBP and MBP-Stau2^62^ were immobilized on different lanes of a SPR sensor chip. GST-hsc70 or GST was injected for 3 minutes over the surfaces in the presence or absence of ATP. The baseline obtained with the MBP-coupled reference surface was subtracted from the sensorgram obtained from the MBP-Stau2^62^-coupled surface and a typical result is shown.

hsc70 contains two functional domains, an N-terminal ATPase domain that contains the ATP/ADP-binding site and a C-terminal peptide-binding domain that contains the substrate-binding pocket [13]. Upon ATP binding and hydrolysis a conformational change is induced in the ATPase domain [14], [15] that modifies the structure of the substrate binding domain and consequently modulates its ability to bind substrates. In its ATP bound form, hsc70 binds and releases substrates quickly, while, in the ADP bound state, substrate binding and release are slow [16], [17]. Therefore, we next tested whether the Stau2^62^/hsc70 association is sensitive to the presence of ATP. To this end, the co-immunoprecipitation experiment (Fig. 4A) and the SPR experiment (Fig. 4B) were reproduced in the presence of 10 mM ATP. In both cases, the presence of ATP almost completely abolished the Stau2^62^/hsc70 interaction.

### Co-localisation of Stau2 isoforms with YB1 and PABPC1 in dendrites of hippocampal neurons

**Figure 5:**
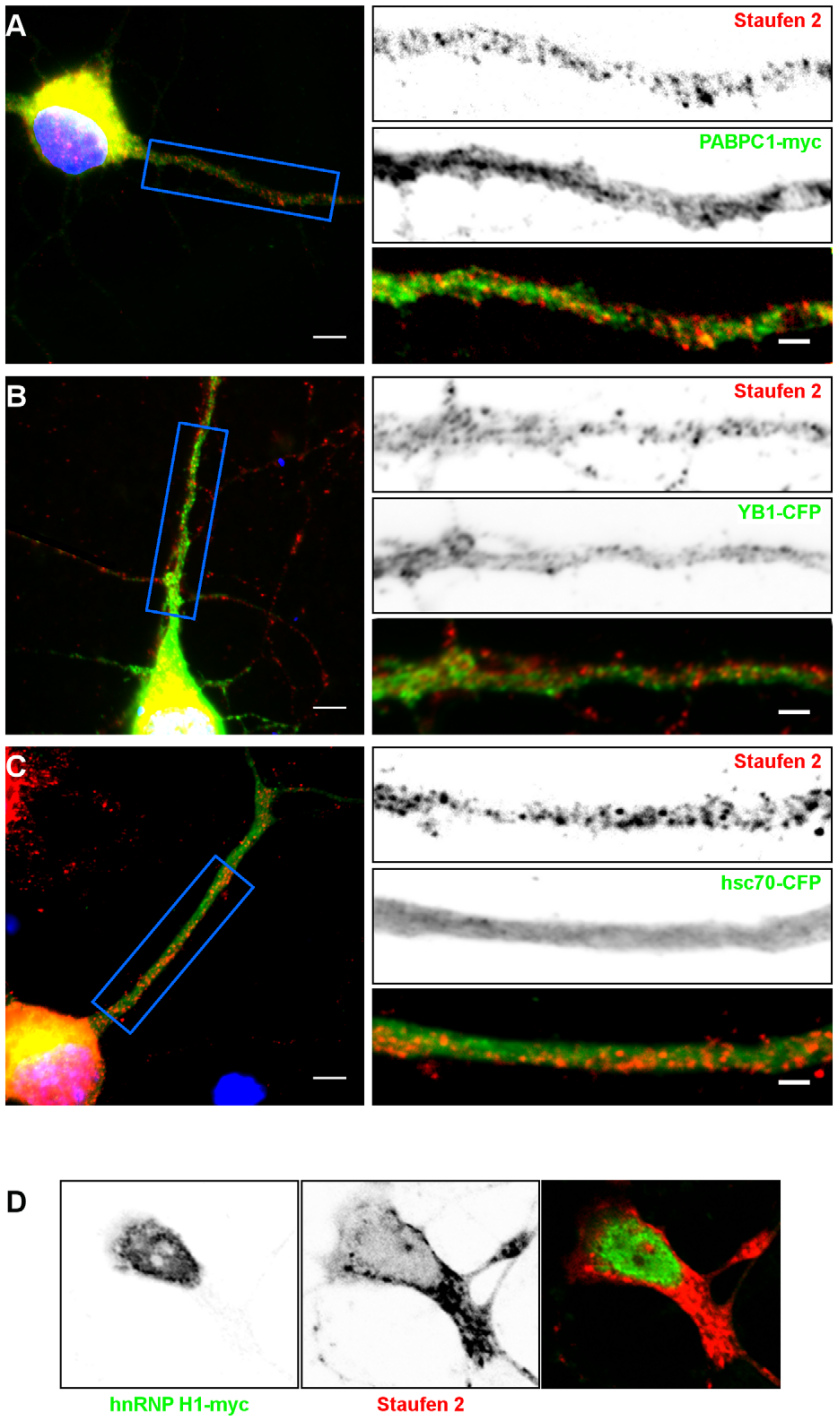
Co-localization of endogenous Stau2 with PABPC1-myc, YB1-CFP, hsc70-CFP and hnRNP H1-myc in hippocampal neurons. Neurons were transfected with plasmids coding for either PABPC1-myc (**A**), YB1-CFP (**B**), hsc70-CFP (**C**) or hnRNP H1-myc (**D**). Twenty four hours post-transfection, neurons were fixed and labeled with anti-myc or anti-GFP (green) and anti-Stau2 (red) antibodies. **Left**: Fluorescence microscopy of hippocampal neurons in culture. Scale bars: 5 µm. **Right**: Higher magnification of images showing protein localization in dendrites. The lower panels represent the superposition of both green and red signals. Scale bars: 2 µm.

Altogether, our data suggest that we have isolated Stau2-containing mRNA particles, a sub-population of all Stau2-associated complexes in brains [6], and that these mRNPs contain YB1, PABPC1 and/or hsc70. To determine whether these proteins can also be detected in the large granule complexes that are visible in dendrites, embryonic hippocampal neurons were first transfected with plasmids coding for PABPC1-myc, YB1-CFP, hsc70-CFP or hnRNP H1-myc and fixed. Tagged-proteins and endogenous Stau2 were detected with anti-myc or anti-GFP and anti-Stau2 antibodies, respectively (Fig. 5). In addition to their presence in the cell bodies, PABPC1-myc (Fig. 5A) and YB1-CFP (Fig. 5B) can be found as puncta in dendrites (Fig. 5A). Only a very small fraction of these puncta also stained with antibodies that recognized endogenous Stau2. This suggests that PABPC1 and YB1 are not components of the large Stau2-containing complexes in dendrites. Similarly, hsc70-CFP was homogeneously distributed in the cell body and dendrites and did not form observable puncta (Fig. 5C). Therefore, it is suggested that its association with Stau2 may occur outside the large dendritic RNP complexes. Finally, hnRNP H1-myc was strictly nuclear (Fig. 5D), indicating that, if confirmed, Stau2/hnRNP H1 association would be restricted to the nucleus.

**Figure 6:**
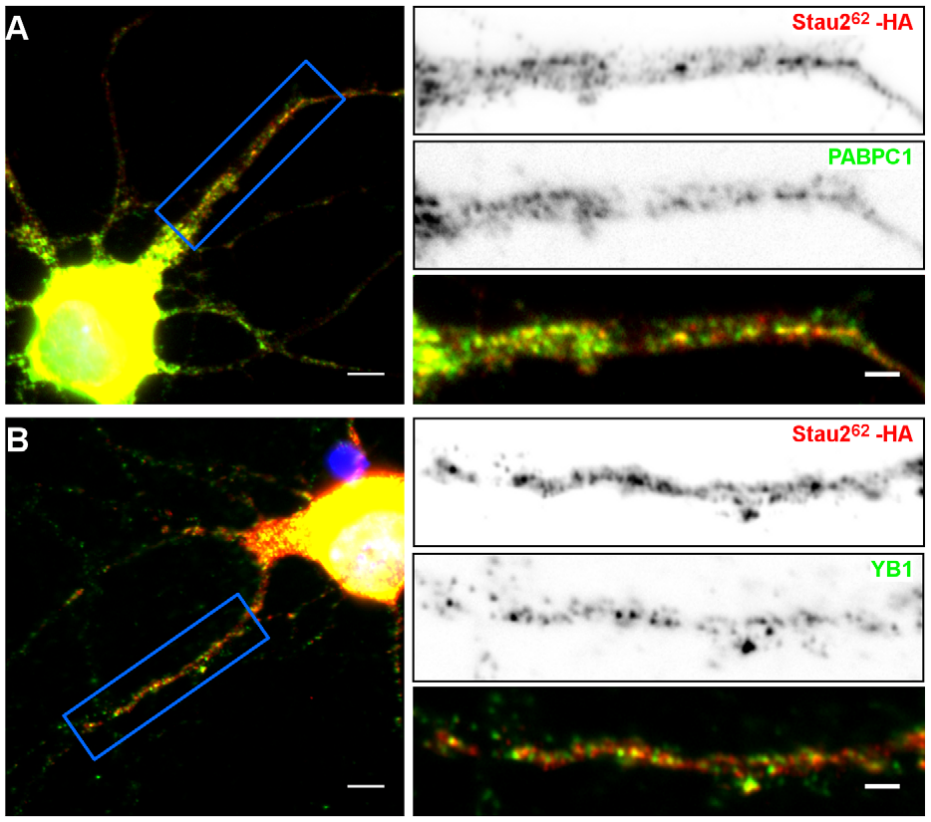
Co-localization of Stau2^62^-HA_3_ with endogenous PABPC1 and YB1 in hippocampal neurons. Neurons were transfected with a plasmid coding for Stau2^62^-HA_3_. Twenty four hours post-transfection, neurons were fixed and labeled with anti-HA antibody (red) and either anti-YB1 (**A**) or anti-PABPC1 (**B**) antibodies (green). **Left**: Fluorescence microscopy of hippocampal neurons in culture. Scale bars: 5 µm. **Right**: Higher magnification of images showing protein localization in dendrites. The lower panels represent the superposition of both green and red signals. Scale bars: 2 µm.

The reverse experiment was also done. Hippocampal neurons were transfected with Stau2^62^-HA_3_ and its co-localization with endogenous PABPC1 (Fig. 6A) and YB1 (Fig. 6B) was analyzed with specific antibodies. In these conditions, PABPC1 partly co-localized with Stau2^62^-HA_3_ whereas YB1 displayed only a weak co-localization. Altogether, our results suggest that PABPC1, YB1, hsc70 and hnRNP H1 may be mostly associated with Stau2 in small mRNPs and mainly absent in the large Stau2-containing mRNA granules in dendrites.

## Discussion

Stau2 is an RNA-binding protein mainly expressed in brain [7] and its importance for mRNA transport in dendrites [8], [18] and synaptic functions [9] has been established. As a further step aiming to define its molecular functions in neurons and the mechanisms of regulation of Stau2-mediated mRNA transport and translation, we determined the molecular composition of Stau2-containing mRNPs. The absence of ribosomal proteins in the proteomics suggests that Stau2-containing particles have been characterized. They mainly contain components of the microtubules and RNA-binding proteins. While PABPC1, YB1 and hsc70 are clearly associated with Stau2-mRNPs, the presence of hnRNP H1 in these complexes is still unclear. It is possible that hnRNP H1 only transiently interacts with Stau2 in the nucleus and/or that it specifically associates with Stau2^56^ or Stau2^52^ isoform(s). Alternatively, hnRNP H1 may be a false positive hit in the proteomics. However, hnRNP H1 has previously been identified in polysome-free poly(A)-bound mRNA complexes [19] and in embryonic RNA granules [20] suggesting that it is a component of at least some mRNPs.

Post-transcriptional regulation of gene expression relies on a highly heterogeneous population of mRNP particles that ensure proper mRNA processing during splicing, nuclear trafficking, cytoplasmic localization, translation and/or decay [21], [22]. The differential presence/absence of RNA-binding proteins and other cofactors in each mRNP determines the roles of each mRNP complex in cellular functions and the fate of associated mRNAs. They may also form organized domains such as nuclear speckles, P-bodies or stress granules that are visible under the microscope when using specific markers [23], [24], [25]. In dendrites of neurons, large ribonucleoprotein complexes shown to be associated with membranes and/or ribosomes can also be observed [2], . Our results at the biochemical and cellular levels are consistent with the possibility that we have isolated and characterized Stau2-containing ribosome-free mRNPs. A minor population of soluble, ribosome-free Stau2-containing complexes was already described in neurons [6], [7]. This population contained all differentially spliced Stau2 isoforms and was enriched with RNAs [6]. Accordingly, in a parallel approach, we also isolated mRNAs from the immunoprecipitated Stau2-containing mRNPs indicating that the proteomically identified proteins are components of complexes that also contain mRNAs [26]. The role of Stau2 and of its protein partners on the fate of associated mRNAs is still unclear. The presence in the proteomics of a nuclear protein (hnRNP H1), a protein and mRNA chaperone also involved in nuclear import/export (hsc70) and proteins that regulate translation initiation (PABPC1 and YB1) suggests that the isolated Stau2-containning mRNPs may be those involved in mRNP formation in the nucleus and/or in post-transcriptional regulation of bound mRNAs (see below). Stau2 was shown to be involved in mRNA transport in cellular processes [6], [8], [18], [27], and based on known functions of its paralog Stau1, it might also be implicated in the control of mRNA stability or translation [28], [29].

Interestingly all Stau2-associated proteins except RUFY3 were previously described in different mRNPs. Indeed, α- and β-tubulin, YB1, PABPC1, hsc70 and hnRNP H1, as well as Stau2, were all present in the heterogeneous populations of mRNA granules isolated from embryonic rat brains [20]. Different combinations of the proteins were also described in other mRNPs [19], [30], [31]. However, they are not universal components of all mRNPs since they were not identified in RNA granules isolated from post-natal rat brains [32] and only PABPC1 and tubulins were found in Stau1-containing mRNPs [33], [34] suggesting that they play specialized roles for the transport and translation of specific mRNAs. In contrast, RUFY3 is specific to Stau2-containing mRNPs. This poorly characterized protein is expressed in brain and peaks around post-natal day 4. It accumulates in growth cones of minor processes and axons. Down-regulation of RUFY3 expression by RNAi leads to an increase in the population of neurons bearing surplus axons [35]. Its molecular function and its role within RNPs are completely unknown.

One of the fundamental questions in the field is how mRNA translation is repressed during transport and reactivated in response to cell needs. It is believed that mRNA transport particles are translationally repressed at the level of initiation whereas ribosome-associated granules are kept silent during elongation [3]. Accordingly, our proteomic results on Stau2-containing RNPs identified YB1 and PABPC1, two proteins known to modulate translation through interaction with initiation factors, as prominent candidates to fulfill translational regulation. YB1 is known to play key roles in cap-dependent mRNA stabilization [36] and translation [37], [38]. It is viewed as a general translational repressor that maintains mRNPs in a translationally silent state by its ability to bind the 5′cap structure thus displacing the initiation factors eIF4E and eIF4G from these mRNAs [36], [37]. Interestingly, YB1 can be phosphorylated by the Akt kinase [39], [40], the Rsk1/2 kinase and PKC alpha [41]. Its phosphorylation by the Akt kinase specifically diminishes its interaction with the capped 5′ mRNA terminus reducing its ability to inhibit cap-dependent translation. Therefore, upon activation of a signalling pathway, phosphorylation of YB1 would be an efficient mechanism to modulate local translation of Stau2-bound mRNAs in neurons. Similarly, PABPC1 is involved in both activation and repression of translation at the level of initiation. By binding simultaneously to the poly(A) tail of mRNAs and to the initiation factor eIF4G, PABPC1 facilitates the formation of the closed loop structure that facilitates translation. PABPC1 is also involved in translation inhibition by repressors [42]. Indeed, poly(A) tail, PABPC1 and eIF4G are all required to allow the translational inhibition of the ceruloplasmin transcript by the IFN-gamma-activated inhibitor of translation (GAIT) repressor. Similarly, PABPC1 is involved in the translational repression of its own transcript by binding an adenine-rich autoregulatory sequence (ARS) in the 5′-untranslated region [43]. It also binds with lower affinities to a non-poly(A) cis-acting dendritic localizer sequence in the vasopressin mRNA [43], [44], [45]. In addition to their roles in translation, both YB1 [46] and PABPC1 [47] strongly bind tubulins. The association between YB1 and tubulin is believed to interfere with mRNA binding thus reducing the YB1/mRNA ratio and facilitating translation. Considering that Stau2 mRNPs are transported on microtubules in dendrites and that both α- and β-tubulin were found in the proteomics, the presence of YB1 and PABPC1 in Stau2-containing mRNPs may contribute to the control of mRNA translation activation by regulating mRNA accessibility.

Another proteomically identified protein is hsc70. Hsc70 has a chaperone activity driven by cycles of ATP/ADP bound states [15]. It could be an important factor for Stau2 folding, thus enabling it to bind mRNAs and/or other protein partners. It may also more directly influence mRNA metabolism and/or translation through its ability to modulate the folding of mRNA [48] and to stabilize mRNA via its binding to AU-rich sequences [49]. Interestingly, its mRNA binding activity is inhibited by the binding of ATP molecules that compete for the same protein domain [48]. We show in this paper that ATP binding also inhibits its interaction with Stau2 (Fig. 4) suggesting that hsc70 through ATP binding may regulate the release of mRNAs and/or control the dissociation of Stau2-containing RNPs. Releasing and/or destabilising mRNAs at precise moments during synaptic activity can finely tune protein expression at synapses. Hsc70 can play two additional important roles in mRNP transport. First, hsc70 is known to regulate the nuclear export/import trafficking of several karyopherin family members [50], [51]. Therefore, it could be an important factor for the nuclear shuttling of Stau2 and/or nuclear exit of Stau2-containing mRNPs. However, its role in the trafficking of exportin-5 and CRM1 which are known to export Stau2 from the nucleus [10], [52] has not yet been tested. Finally, hsc70 is important for the release of the molecular motor kinesin from its vesicular cargo permitting its precise localization [53]. Stau2 mRNPs are transported on dendritic microtubules [7] and co-fractionate with tubulin and kinesin [6] making hsc70 an important candidate for the regulation of their transport. Altogether, these functions of hsc70, especially those related to protein and mRNA folding and to nuclear export/import, suggested that hsc70 is involved in mRNP formation and/or other early steps in the process of mRNA transport. This may explain why the presence of hsc70 in large Stau2-containing mRNA transport complexes in dendrites may not be required (Fig. 5).

In conclusion, our data suggest that we have characterized a heterogeneous population of Stau2-containing mRNPs from embryonic rat brain. Our study indicates that they are largely composed of proteins that are also components of other types of mRNPs. The biochemical characterization of Stau2-containing particles gives us new tools that will contribute to our understanding of mRNA transport in neurons and of its role in neurons.

## Materials and Methods

### Ethics statement

Pregnant Sprague-Dawley rats were purchased from Charles Rivers Canada. Our research involving animals has been conducted according to the guidelines of the Canadian Council of Animal Care (CCAC). Our project has been approved by the “Comité de déontologie de l′expérimentation sur les animaux” at the Université de Montréal.

### Immunoprecipitation and immunoblotting

Rabbit polyclonal anti-Stau2 [7] and a mouse monoclonal [29] anti-HA antibodies were used for immunoprecipitation. For immunoblotting, mouse monoclonal anti-Stau2 [7], rabbit polyclonal anti-HA (Sigma), goat anti-myc (Bethyl), goat anti-GST (Amersham Pharmacia Biotech), goat anti-GFP (Rockland), and rabbit anti-MBP (New England BioLabs, Inc) were used. For immunofluorescence in hippocampal neurons, rabbit polyclonal anti-Stau2 (a generous gift from Dr Michael Kiebler), anti-PABPC1 (Abcam) and anti-YB1 (Abcam) were used.

Immunoprecipitation of Stau2-containing mRNPs was performed on cell extracts prepared from whole brains of E17–E18 rat embryos. Cells were dissociated with a manual putter and lysed in 50 mM Tris-HCl (pH 7.5), 100 mM NaCl, 0.5% Triton X-100, 15 mM EGTA, 1 mM DTT and complete EDTA-free protease inhibitor cocktail (Roche). Cell lysates were centrifuged at 9300 g for 10 min to remove nuclei and cell debris. After centrifugation, supernatants were incubated with a rabbit polyclonal anti-Stau2 antibody [7] for 2 hours at 4°C, then with a 50% protein A-sepharose slurry for 2 hours at 4°C. Immune complexes were washed five times with the lysis buffer and eluted from the resin by heating at 95°C for 5 minutes in elution buffer (100 mM Tris-HCl pH 7.4, 200 mM DTT and 4% SDS). RNAs were isolated by Trizol (Invitrogen) extraction.

For the co-immunoprecipitation experiments, N2A cells were propagated in DMEM medium supplemented with 10% BSA serum (HyClone). Cells were co-transfected with plasmids coding for one of the following proteins: Stau2^59^-HA_3_ or Stau2^62^-HA_3_ [12] and either hsc70-CFP, YB1-CFP, hnRNP H1-myc or PABPC1-myc using the calcium-phosphate technique. Cells were collected 48 h post-transfection. Immunopurifications were performed as above using a mouse monoclonal anti-HA antibody. Detection of the co-immunoprecipitated proteins was done by western blotting with either rabbit polyclonal anti-HA (Sigma), goat anti-Myc (Bethyl) or goat anti-GFP (Rockland) antibodies. To asses if the interaction was RNA dependent, cell extracts were incubated for 30 min at room temperature with 300 U (300 U/ µl) of the Micrococcal Nuclease (Fermentas) in 1 mM CaCl2 before adding the antibody.

### Proteomic techniques

Proteomic techniques were essentially done as before [20]. Briefly, immunoprecipitation eluates were separated by SDS-PAGE, stained with Coomasie Blue, and cut into 26 horizontal gel slices with each slice processed for in-gel trypsin digestion and peptide extraction. The extracted peptide mixtures were separated and analyzed in an automated system by nanoscale LC Q-TOF MS/MS. After fragmentation in the MS/MS mode, the resulting spectra were searched with Mascot (version 1.9.03; Matrix Science, London, UK) against a copy of the National Center for Biotechnology Information (NCBI) non-redundant protein database (June 21^st^, 2004) restricted to the *Mammalia* taxonomy.

### Protein co-localisation in hippocampal neurons

Primary hippocampal neurons were cultured on #1,5 coverslips as previously described [12], [54]. On day 5, neurons were transfected with 2 µg of Lipofectamine™ 2000 and 1 µg of plasmids coding for Stau2^62^-HA_3_, PABPC1-myc, hsc70-CFP, YB1-CFP, or hnRNP H1-myc as indicated in 100 µl of plain Neurobasal for 10 min. Neurons were fixed 24 hours later with PBS/PFA 4%, PFA was quenched with 1 M glycine in PBS/0.1% Triton X-100 for 10 min, blocked with 0.1% Triton X-100/2% BSA in PBS overnight at 4°C. Neurons were incubated with goat anti-myc (1∶400 Bethyl A190–104A), mouse anti-GFP (1∶500, Roche) or anti-HA (1∶3000, 12CA5) and rabbit anti-Stau2 (1∶600, a generous gift from Dr Michael Kiebler), rabbit anti-PABP or anti-YB-1 (1∶200 and 1∶250, Abcam ab21060 and ab12148, respectively) antibodies for 2 h at room temperature, washed in PBS and stained with Alexa Fluor®647 dyed donkey anti-rabbit immunoglobulin G (IgG) and Alexa Fluor®488 dyed donkey anti-mouse or anti-goat IgG antibodies (1∶400, Invitrogen A31573, A21202 and A11055, respectively) for 1 h. Coverslips were mounted on slides (Fisher) using Dako fluorescent mounting medium (Dako). Neurons were visualized under a Nikon E800 widefield microscope Plan Apo 100× N.A. 1.40 oil-immersion objective lens.

### Protein expression and purification

Plasmids coding for GST-hsc70 and MBP-Stau2 were cloned into the pGEX (Amersham Pharmacia Biotech) and pMal-C (New England Biolabs) vectors, respectively. The fusion proteins were expressed in *E. coli* BL21 cells, following induction with IPTG for 2 h. Proteins were collected in PBS/1 mM DTT/1% Triton X-100/5 mM benzamidine at 4°C. GST-hsc70 and MBP-Stau2 proteins were purified on glutathione-Sepharose-4B and amylose affinity columns, respectively and eluted with 10 mM reduced glutathione in 50 mM tris-HCl (pH 8.0) or 10 mM of D-maltose in PBS, respectively. For some experiments, lysates were treated with 50 µg/ml RNAse A for 1 h at 4°C before column purification. Protein purification was monitored by SDS-PAGE. Proteins were detected by zinc staining and/or western blotting experiments.

### GST-pull down assay

Bacterially expressed and purified GST and GST-hsc70 proteins were attached to glutathione columns. MBP-Stau2 or MBP were loaded onto the columns, extensively washed and eluted as recommended by the manufacturer. Eluted proteins were analyzed by western blotting using anti-GST and anti-MBP antibodies. For some experiments, protein extracts were treated with 50 µg/ml RNAse A for 1 h at 4°C before loading onto the columns.

### Surface plasmon resonance (SPR) binding assay

Binding interactions between purified MBP-Stau2 and GST-hsc70 were examined in real time using a BIACORE 2000 instrument (GE Healthcare Bio-Sciences AB, Upssala, Sweden). Experiments were performed on research-grade CM5 sensor chip at 25°C using filtered (0.2 µm) and degassed HBS-EP [10 mM Hepes pH 7.4, 150 mM NaCl, 3 mM EDTA, 0.005% (v/v) Surfactant P20]. Protein-grade detergents [10% (v/v) Tween-20, 10% (v/v) DDM] were from Calbiochem; all other chemicals were reagent grade quality. Immobilized sensor chip surfaces were prepared using the Biacore amine Coupling Kit. Briefly, 35 µl of a freshly mixed solution of 200 mM 1-ethyl-3-(3-dimethylaminopropyl)-carbodiimide and 50 mM N-hydroxysuccinimide was injected (at a flow rate of 5 µl min^−1^) to activate surface-exposed carboxymethyl groups into reactive esters. Next, 120 µl of MBP-Stau2 diluted to 70 µg ml^−1^ in 10 mM sodium acetate pH 4.0 was injected (at a flow rate of 10 µl min^−1^) to generate amine-coupled protein surfaces. Finally, 70 µl of 1 M ethanolamine pH 8.5 was injected to deactivate excess reactive groups and remove any non-specifically bound ligand. A reference surface was prepared in a similar manner with purified MBP. To test binding, purified GST-hsc70 (60 µg ml-1) or GST (negative control) were injected over the coupled surfaces at 10 µl min^−1^ (3 min association time and 2.5 min dissociation time). For all SPR assays, the surfaces were regenerated between sample injections at 50 µl min^−1^ with a 30 s single pulse of 0.4 M NaCl followed by a stabilization time after regeneration of 3 min. The assays were performed in duplicates with different batches of purified proteins.

## Supporting Information

Table S1 Proteomically identified proteins in Stau2-containning mRNPs.
(0.05 MB DOC)
