# Supplementary material for: Molecular Composition of Staufen2-Containing Ribonucleoproteins in Embryonic Rat Brain
Source: PLoS One. 2010 Jun 28;5(6):e11350. doi: 10.1371/journal.pone.0011350 (PMC2893162; doi:10.1371/journal.pone.0011350)
Supplement: Table S1 — Proteomically identified proteins in Stau2-containning mRNPs. (0.05 MB DOC) [file pone.0011350.s001.doc]

**Table S1**

| ***Identified proteins*** | ***Ref :*** | ***Peptides matched*** | ***Mol mass*** | ***Gel band #*** | ***Mascot score*** |
| --- | --- | --- | --- | --- | --- |
| Polyadenylate-binding protein, cytoplasmic 1 (Pabpc1) | [NP_599180.1](https://portal.proteomics.mcgill.ca/http:/mmc.protsearch.mcgill.ca/mascot/cgi/#Hit1) | AKEFTNVYIK  ALDTMNFDVIK  KEFSPFGTITSAK  NLDDGIDDERLR  GFGFVCFSSPEEATK  SKVDEAVAVLQAHQAK  SLGYAYVNFQQPADAER | 73 kDa | 16 | 117 |
| Heat shock protein 8 (hspa8; hsc70) | [NP_077327.1](https://portal.proteomics.mcgill.ca/http:/mmc.protsearch.mcgill.ca/mascot/cgi/#Hit2) | MKEIAEAYLGK  ARFEELNADLFR  NQVAMNPTNTVFDAK | 71 kDa | 16 | 57 |
| heterogeneous nuclear ribonucleoprotein H1 (hnrnph1) | [NP_543172.1](https://portal.proteomics.mcgill.ca/http:/mmc.protsearch.mcgill.ca/mascot/cgi/#Hit22) | STGEAFVQFASQEIAEK | 50 kDa | 12 | 75 |
| Y box-binding protein 1 (Yb1) | [NP_113751.1](https://portal.proteomics.mcgill.ca/http:/mmc.protsearch.mcgill.ca/mascot/cgi/protein_view.pl?file=/proline/data06/mascot/data/../data/20040625/F172441.dat&hit=gi|112410&px=1&protscore=97.3) | GAEAANVTGPGGVPVQGSK  NDTKEDVFVHQTAIK  PQYSNPPVQGEVMEGADNQGAGEQGRPVR | 36 kDa | 11 | 97 |
| RUN and FYVE domain containing protein 3 (RUFY3)  (Rap2 interacting protein x; [Ripx;](http://www.ncbi.nlm.nih.gov/sites/entrez?db=gene&cmd=search&term=360921&RID=HNZSNBDF01R&log$=geneexplicitprot&blast_rank=1) Singar1) | [NP_001020298.1](https://portal.proteomics.mcgill.ca/http:/mmc.protsearch.mcgill.ca/mascot/cgi/protein_view.pl?file=/proline/data06/mascot/data/../data/20040625/F172445.dat&hit=gi|17160984&px=1&protscore=60.2371911660722) | GSEGDGQITAILDQK  NYVEELNR  LTEELAVANNR  IITLQEEMER  LVPEAAEITASVK  VSMDGEWLCLR  VKEESSYLLESNR  LDVEKELELQISMR  ELDDISLTPDPEPTHEDPNYLMANER  HLNATVNNLQAK | 53 kDa | 13-14-15 | 312 |
| Tubulin, beta 2b (Tubb2b) | [NP_001013908.2](http://portal.proteomics.mcgill.ca/portal/proxy/mmc/mascot/cgi/protein_view.pl?file=/proline/data06/mascot/data/../data/20040625/F172442.dat&hit=gi|21746161&px=1&protscore=389.402811102342&_mudpit=1000) | FPGQLNADLR  LAVNMVPFPR  ISEQFTAMFR  INVYYNEATGNK  AILVDLEPGTMDSVR  LHFFMPGFAPLTSR  NSSYFVEWIPNNVK  ALTVPELTQQMFDSK  MSATFIGNSTAIQELFK  GHYTEGAELVDSVLDVVR  GHYTEGAELVDSVLDVVRK  MREIVHIQAGQCGNQIGAK | 55 kDa | 12 | 389 |
| Tubulin, alpha 1a (Tuba1a) or Tubulin, alpha 1b (Tuba1b) | [NP_071634.1](http://portal.proteomics.mcgill.ca/portal/proxy/mmc/mascot/cgi/protein_view.pl?file=/proline/data06/mascot/data/../data/20040625/F172443.dat&hit=gi|37492&px=1&protscore=135.01&_mudpit=1000)  NP_001037735.1 | QLFHPEQLITGK  AVFVDLEPTVIDEVR  NLDIERPTYTNLNR  VGINYQPPTVVPGGDLAK  TIGGGDDSFNTFFSETGAGK | 55 kDa | 13 | 135 |
| Staufen 2 (Stau2) | [NP_001007150.1](http://portal.proteomics.mcgill.ca/portal/proxy/mmc/mascot/cgi/protein_view.pl?file=/proline/data06/mascot/data/../data/20040625/F172444.dat&hit=gi|19526444&px=1&protscore=347.227191166072&_mudpit=1000) | NMPVSFEVIK  IQGFQAALSALK  VTSGTTLGYLSPK  NAAEAMLLQLGYK  ALQALQNEPIPEK  AGPEYGQGMNPISR  VSVGEFSAEGEGNSK  ELLMNGTSPAAEAIGLK  GEPAIYRPLDPKPFPNYR  SNVNNNPGSITPTVELNGLAMK  SNVNNNPGSITPTVELNGLAMKR  DMNQPSSSFFSVESPSPTSSAPAAR | 59-63 kDa | 14 | 347 |

Proteomically identified proteins in Stau2-containning mRNPs
